# Diet-Attributable Greenhouse Gas Emissions and Acute Myocardial Infarction in Costa Rica Heart Study

**DOI:** 10.3390/nu16010138

**Published:** 2023-12-30

**Authors:** Abeer A. Aljahdali, Hannia Campos, Keylin Granados, Andrew D. Jones, Ana Baylin

**Affiliations:** 1 Department of Clinical Nutrition, Faculty of Applied Medical Sciences, King Abdulaziz University, Jeddah 21589, Saudi Arabia; aaoaljahdali1@kau.edu.sa; 2 Department of Nutritional Sciences, University of Michigan, Ann Arbor, MI 48109, USA; jonesand@umich.edu; 3 Center for Research and Innovation in Translational Nutrition, Universidad Hispanoamericana, San Jose 10101, Costa Rica; hacanu@gmail.com (H.C.); keylingranados@gmail.com (K.G.); 4 Department of Nutrition, Harvard T. H. Chan School of Public Health, Boston, MA 02115, USA; 5 Department of Environmental Health Sciences, University of Michigan, Ann Arbor, MI 48109, USA; 6 Department of Global Public Health, University of Michigan, Ann Arbor, MI 48109, USA

**Keywords:** sustainable food consumption, red meat, diet-attributable GHGEs, cardiovascular diseases, Costa Rican adults, population-based case–control study

## Abstract

Adopting sustainable dietary patterns is essential for planetary and human health. As data to address this issue are lacking in Latino populations, this study examined the association between diet-attributable greenhouse gas emissions (GHGEs) and myocardial infarction (MI) in a Costa Rica Heart Study. This analysis included 1817 cases of a first non-fatal acute MI during hospitalization and their matched population-based controls, by age, sex, and area of residence. A validated food frequency questionnaire was used to quantify habitual dietary intake and diet-attributable GHGEs (kg CO_2_ equivalent (eq.)/year). Due to the matching design, conditional logistic regression was used. Red meat consumption contributed approximately 50% to the total diet-attributable GHGEs among both cases and controls. Higher diet-attributable GHGEs were associated with increased odds of acute MI. The odds of MI were 63% higher (OR = 1.63; 95% CI 1.20–2.21) among participants in the highest quintile (median diet-attributable GHGEs = 6247 kg CO_2_ eq./year) compared to the lowest quintile (median diet-attributable GHGEs = 2065 kg CO_2_ eq./year). An increasing linear trend in the odds of acute MI and diet-attributable GHGEs was detected (*p*-trend 0.0012). These findings highlight the importance of reducing red meat consumption to sustainably mitigate the incidence of MI and improve planetary health.

## 1. Introduction

The global food system is facing challenges in feeding the growing world’s population [1] and mitigating climate change [2]. The food system is responsible for soil degradation, water depletion, and greenhouse gas emissions (GHGEs) [3], contributing to a third of the total GHGEs [4,5], which cause climate change. Therefore, adopting a sustainable food consumption pattern is crucial to achieving the goals of the Paris 2015 Agreement of keeping global temperature increases at less than 2 °C to curb climate change’s negative consequences [6,7,8,9]. Modifying intake to meet sustainability goals could be accomplished via integrated efforts on the supply side (i.e., efficient production, transporting, and processing) and the demand domain (i.e., adherence to a healthy and sustainable diet and reducing food loss and waste) [9].

A sustainable diet aims to maintain human and environmental health using an affordable, economically fair, safe, and culturally acceptable diet [10]. The characteristics of a sustainable diet are not well-defined since its definition should be context-specific as it is influenced by sociocultural, economic, and other factors [8,11,12,13,14,15,16,17,18]. However, scholars agree that a sustainable diet should include a higher intake of plant-based foods while lowering the intake of animal products (i.e., red/processed meat and cheese) and foods high in fat or sugar [12]. Because the production of plant-based food accounts for 29% of global total diet-attributable GHGEs compared to 57% for animal food production [4], plant-based food is a pillar in sustainable eating practices. In 2019, the EAT Lancet Commission used the concept of a safe operating space for humanity with planetary boundaries [19] to propose a framework for food systems that could provide healthy diets aligned with the environmental sustainability goals to feed the global population of 10 billion people by 2050 [7]. 

Human participation via food choices is vital to achieve environmental and health benefits, and the public should be encouraged to adopt sustainable food habits. In fact, evidence linking a sustainable diet and health benefits is needed to promote sustainable dietary patterns [20]. As a result, multiple studies have examined the association between sustainable dietary practices and health outcomes. Nevertheless, the available findings are not consistent and the majority were focused on mortality [14,21,22,23,24,25,26,27], with no emphasis on short-term benefits. Furthermore, the current literature is mostly limited to high-income countries [21,22,23,24,25,26], so evidence from lower–middle-income countries is needed [28] to acknowledge the context-specific characteristics of a sustainable diet [8,11,12,13,14,15].

Costa Rica—a middle-income country—has experienced a nutrition transition evidenced by changes in dietary intake and activity patterns and a higher prevalence of diet-related chronic diseases over the last few decades [29,30,31,32,33,34,35]. During the nutrition transition, Costa Ricans reported lower red meat intake compared to Western countries [36]; thus, it is worth investigating if sustainable food choices still confer health benefits with low red meat consumption. Therefore, we analyzed the associations between a sustainable diet, assessed using diet-attributable GHGEs, and myocardial infarction (MI) among the Costa Rica Heart Study’s participants between 1994 and 2004. We hypothesized a positive relationship between the risk of acute MI and diet-attributable GHGEs because red meat is a driver of diet-attributable GHGEs [4] and has been shown to be associated with cardiovascular diseases [37,38,39,40,41]. If our hypothesis is confirmed, our findings will further support the potential of individual food choices to confer health and environmental benefits in a middle-income country. 

## 2. Materials and Methods

### 2.1. Study Population

The subjects in this study were participants in the Costa Rica Heart Study, a population-based case–control study of adults aged 20 to 70 years, explicitly designed to evaluate diet and heart disease. Details of the study have been reported elsewhere [42]. The catchment area comprised 7071 km^2^ and 2,057,000 people, ethnically Mestizo (as a result of four centuries of tripartite mixing—European, African, Amerindian) and culturally Hispanic American [43] and covered a full range of socioeconomic levels and urban, peri-urban, and rural lifestyles. Eligible incident cases were men and women who were survivors of a first acute MI as diagnosed by a cardiologist at any of the 6 recruiting hospitals between 1994 and 2004. The hospitals were visited daily by the study fieldworkers, and cases were confirmed by two independent cardiologists according to the World Health Organization established criteria for MI at the moment of recruitment (1994–2004), which include either an increase in cardiac enzymes or diagnostic-related changes in electrocardiogram and typical MI symptoms [44]. Enrollment was carried out while cases were in the hospital’s step-down unit. Cases were excluded if they died during hospitalization, were ≥75 years of age on the day of their first MI, were physically or mentally unable to answer the questionnaire, or had a previous hospital admission related to cardiovascular disease. Each incident case matched (1:1) for age (±5 years), sex, and area of residence (county) was randomly selected using information available from the National Census and Statistics Bureau of Costa Rica. Controls were ineligible if they were physically or mentally unable to answer the questionnaires or if they had ever had an acute MI. Participation was 98% for cases and 88% for controls. All subjects gave informed consent on documents approved by the Human Subjects Committee of the Harvard School of Public Health and the University of Costa Rica.

### 2.2. Data Collection

Trained fieldworkers collected all data during an interview using a standardized questionnaire consisting of closed-ended questions that inquired about information such as current smoking status (yes/no); sociodemographic, including marital status (married or not), education (completed 14 years or more of education or not), and household income ($ US/month); and medical history for diabetes and hypertension (yes/no) [42]. Waist circumference (cm) was collected in duplicate from subjects in light clothing. Dietary intake was collected using a 135-item semi-quantitative food-frequency questionnaire (FFQ) specifically developed and validated to assess dietary intake during the past year in the Costa Rican population [45]. Subjects were asked to choose one of nine categories of food intake: never or less than/month, 1–3/month, 1/week, 2–4/week, 5–6/week, 1/day, 2–3/day, 4–5/day, or 6 or more/day. For cases, average intake represented the year preceding their MI. Intakes of nutrients were calculated using the US Department of Agriculture food composition data file and analysis of Costa Rican foods [46]. Physical activity was assessed using a questionnaire about the frequency and time spent on several occupational and leisure activities during the last year. The total metabolic equivalents (METs/day) were calculated by summing the METs for all physical activities in the questionnaire [47,48].

### 2.3. Diet-Attributable GHGEs

To estimate GHGEs, we matched each food item listed in our FFQ with a diet-attributable GHGEs database by Poore and Nemecek [49]; in this dataset, the diet-attributable GHGEs were calculated using life cycle analysis data from 570 studies conducted in 119 countries around the globe, including Costa Rica, and included all stages of food production and post-farm activities such as packaging and retail [49]. After matching the FFQ items, we multiplied the mean in kg CO_2_ equivalent (eq.) per 100 g in the Poore and Nemecek dataset [49] by the portion size of each food item in the FFQ. Then, the diet-attributable GHGEs per year were estimated by multiplying the GHGEs for each food item by the frequency of food intake (times per day) multiplied by 365. The total diet-attributable GHGEs were calculated and expressed in a kilogram of CO_2_ eq./year. We calculated the group-specific diet-attributable GHGEs after categorizing the FFQ food items into fourteen food groups: vegetables, fruits, grains, cereals and starchy vegetables, nuts and legumes, red meat, fish and chicken, dairy, eggs, sugar, unsaturated fat and oils, alcohol, coffee, chocolate, and water, tea, and other beverages.

### 2.4. Statistical Analysis

**Figure 1:**
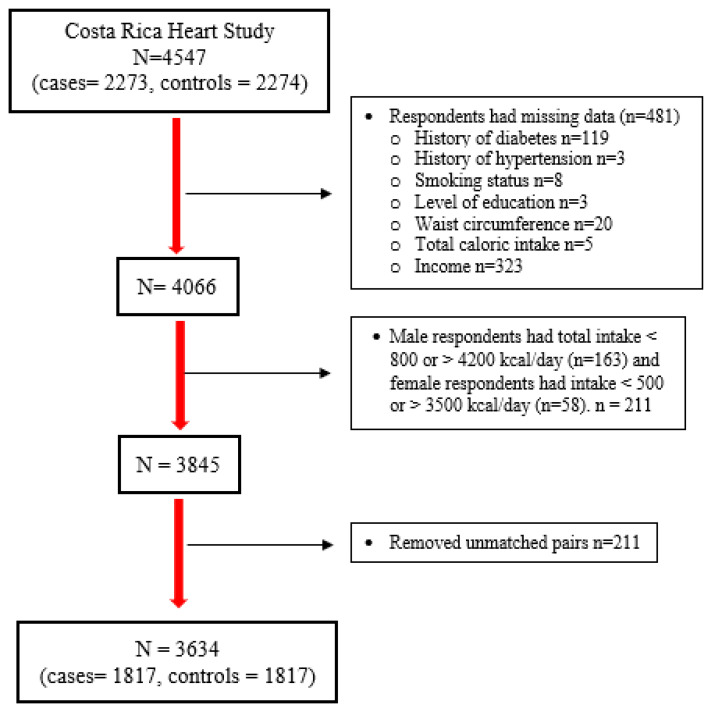
Flowchart summary of analytical samples of the Costa Rica Heart Study.

A total of 1817 case–control pairs were included from the original sample size of 2273 cases and 2274 controls. Subjects with missing data on potential confounders or major explanatory variables and those reporting implausible caloric intake (total caloric intake of <800 or >4200 kcal/day for men, and <500 or >3500 kcal/day for women) [50] were excluded. Subjects were rematched using the original Costa Rica Heart Study matching criteria to avoid losing more sample size. After rematching, any unmatched pairs were excluded (Figure 1). Continuous variables were reported as means and standard deviations, and binary and categorical variables were reported as percentages and frequencies. Significant case–control differences in binary variables were examined with McNemar’s test. Significant case–control differences for normally distributed continuous variables were tested by paired two-tailed *t*-tests, while non-normally distributed continuous variables were tested by Wilcoxon’s Signed Rank test. The distributions of general characteristics, potential confounders, and dietary variables among controls were examined by quintiles of diet-attributable GHGEs.

Odds ratios (ORs) and 95% confidence intervals (CIs) of acute MI associated with a quintile-based increase in diet-attributable GHGEs were estimated using conditional logistic regression models to account for matching between cases and population-based controls, by age, sex, and area of residence. *p*-values for trends across quintiles of diet-attributable GHGEs were computed by assigning the median intake values of each quintile as a continuous variable in the model. Quintile-based categorization of the exposure variables was used instead of the original continuous variables to evaluate dose–response relationships and potential non-linear associations. Confounders were examined and selected based on a priori knowledge and their association with the exposure among the controls. All analyses were performed with Statistical Analysis Systems (SAS) software, version 9.4 (SAS Institute Inc., Cary, NC, USA), and all *p*-values presented were two-sided, with a *p*-value < 0.05 considered statistically significant. 

## 3. Results

Our analysis included 1817 case–control pairs of the participants of the Costa Rica Heart Study (N = 3634), and the average age was 58 years (18–86 years). Table 1 shows the sociodemographic characteristics of the study population stratified by MI status. The mean (SD) age (years) was 58 (11) and 59 (11), for cases and control, respectively, and 57 (11) and 62 (11), for males and females, respectively. The proportion of current smokers and history of diabetes and hypertension was significantly higher in cases compared to controls. On the other hand, education level, income, and physical activity were lower in cases than in controls. There was a significant difference in the mean (SD) diet-attributable GHGEs among cases and controls, respectively, of 4238 (1811) and 3795 (1638) kg CO_2_ eq./year (*p*-value < 0.0001). Similarly, cases had higher mean energy intake than controls (Table 1).

**Figure 2:**
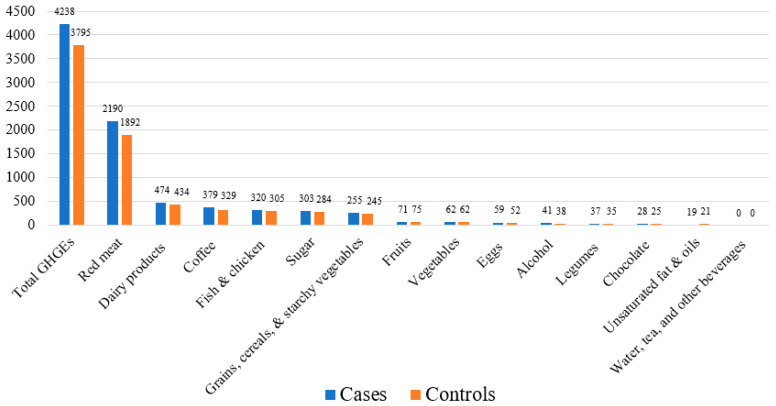
Mean diet-attributable GHGEs (kg CO_2_ eq./year) in the Costa Rica Heart Study (N = 3634).

The mean total and group-specific diet-attributable GHGEs are shown in Figure 2. Red meat had the highest diet-attributable GHGEs compared to the other food groups. Cases had higher mean diet-attributable GHGEs for all food groups, except for the fruit group, which had slightly higher emissions among controls (75 kg CO_2_ eq./year) compared to cases (71 kg CO_2_ eq./year). No group differences were detected in vegetables along with unsaturated fat and oils groups (Figure 2). The food group contributing most to the diet-attributable GHGEs was red meat, which accounted for ~50% of the total diet-attributable emissions, followed by dairy products (12%). The lowest contribution (only 12%) was from fruits, vegetables, legumes, grains, cereals, and starchy vegetables (Table 2). 

The sociodemographic and lifestyle characteristics among controls by quintiles of diet-attributable GHGEs are shown in Table 3. A positive trend was observed between total diet-attributable GHGEs and smoking, income, waist circumference, and total energy intake, whereas an inverse association was observed with a history of hypertension. In addition, percentages for marital status, education level, and history of diabetes differed across the diet-attributable GHGEs quintiles. The group-specific emissions across the total diet-attributable GHGEs quintiles among controls are shown in Table 4. A positive trend for all group-specific emissions was detected across the diet-attributable GHGEs quintiles; however, red meat had the most significant change from the fifth to the first quantile, with an increase of 645%.

The model, adjusted only for matching variables, showed a significant positive trend for acute MI for diet-attributable GHGEs (Table 5). Participants in the highest quintile (median diet-attributable GHGEs = 6247 kg CO_2_ eq./year) had higher odds of acute MI (OR = 2.20, 95% CI 1.78–2.75, *p*-trend < 0.0001) compared to those in the lowest quintile (median diet-attributable GHGEs = 2065 kg CO_2_ eq./year). In the multivariable models, the association between diet-attributable GHGEs and odds of acute MI remained statistically significant, although the point estimates were attenuated. The odds of MI were 1.63 times higher (OR = 1.70, 95% CI 1.20, 2.21) among participants with the highest diet-attributable GHGEs compared to the reference group. There was a significant positive linear trend between the odds of acute MI and diet-attributable GHGEs (*p*-trend = 0.0012). Moreover, for every increase of 1000 kg CO_2_ eq./year, the odds of MI were 1.10 times higher (95% CI 1.04, 1.17) when the diet-attributable GHGEs level was analyzed as a continuous variable.

## 4. Discussion

Using a diverse population of adults in Central Valley, Costa Rica, the association between diet-attributable GHGEs was estimated from an FFQ and the first non-fatal acute MI case–control population-based study. Our data showed that approximately half of the diet-attributable GHGEs were due to red meat consumption. In the current study, our adjusted conditional logistic regression models showed a positive relationship between diet-attributable GHGEs and MI, independent of other sociodemographic correlates. A positive dose–response relationship was detected for higher diet-attributable GHGEs. Our study corroborates the existing knowledge linking a sustainable diet with positive health outcomes, using evidence from a Hispanic/Latino population. 

There are several strengths of this study. First, because of the comprehensive social services provided in Costa Rica, all persons living in the catchment area had access to medical care without regard to income. As a result, control subjects came from the source population that gave rise to the cases and are not likely to have had a cardiovascular disease that was not diagnosed because of poor access to medical care. Therefore, our population-based study design is unlikely to suffer from selection bias. Moreover, our population represents the diverse population residing in the Central Valley of Costa Rica during the nutritional transition, which allows us to generalize the conclusion to the whole population [39]. Finally, we used the diet-attributable GHGEs dataset that combined evidence from 570 studies conducted in 119 countries, including Costa Rica, to estimate the dietary emissions from the farm and post-farm activities (i.e., processing, packaging, and retail) [49]. 

The positive association between diet-attributable GHGEs and MI observed among Costa Rican adults aligns with previous studies conducted among European populations investigating the link between sustainable diet, mortality, and chronic disease burdens [14,21,22,23,24,25,26,27]. Some of these studies investigated the associations with the incidence of coronary heart disease [22], while others included few cardiometabolic biomarkers [24]. These studies showed that higher diet-attributable GHGEs were positively associated with mortality [21,22] and incidence of coronary heart disease [22], while a predominantly plant-based diet effectively reduced premature mortality related to dietary intake worldwide [14]. Similarly, a sustainable reference diet—EAT-Lancet—had favorable associations with the risk of hospitalization or death from ischemic health disease and diabetes, in addition to the beneficial cross-sectional association between EAT-Lancet and cardiometabolic profile [24]. Furthermore, a reduction in the disability-adjusted life years was reported as a benefit for a diet with lower diet-attributable GHGEs [25,26] and a significant reduction in all-cause mortality was detected either by eliminating a third of daily meat intake or substituting it with fish or plant-based foods [23]. On the other hand, other studies reported a null association for total diet-attributable GHGEs or EAT-Lancet on mortality [23,24,27] or the risk of hospitalization or death from stroke [24]. Further studies are needed to fully assess the short-term health-related outcomes for prompting sustainable dietary patterns across different populations. 

Among the study population, red meat consumption accounted for about 50% of the total diet-attributable GHGEs, followed by dairy products (12%) and fish and poultry (10%). On the contrary, fruit, vegetables, legumes, and grains combined contributed to only 12% of the total diet-attributable emissions. Identifying red meat as a driver of the diet-attributable GHGEs is consistent with the findings from other studies [16,17,21,22,23,51,52,53,54,55]. However, cultural differences were noted as a key factor describing the food groups accounting for the highest diet-attributable GHGEs across populations [16,52,56,57]. For example, among Finnish women, dairy products are the main contributor to diet-attributable GHGEs [16]. Among Japanese adults, fish and seafood together and cereals are the second and third contributors of total diet-attributable GHGEs [52]. Rice is the highest contributor to the diet-attributable GHGEs among Chinese [56,57]. As a result, it is crucial to consider the context when assessing the sustainability metrics of consumption. 

It is known that animal food production accounts for 57% of the total diet-attributable GHGEs and beef production accounts for 25% of total animal GHGEs [4]. Also, we showed that red meat is the driver of the diet-attributable GHGEs among our population. Therefore, our positive association between diet-attributable GHGEs and MI could be explained in light of the existing literature associating red meat consumption with cardiovascular diseases [37,38,39,40,41] and plant-based diet with favorable cardiovascular outcomes [58,59,60,61,62]. However, we acknowledge the lack of consideration of the control for temperature variability across seasons and years in the current analysis because existing knowledge has shown that climate change itself has negative health consequences [63,64,65], including cardiovascular outcomes [65,66,67,68,69] and MI [70,71,72,73]. Given that the recruitment period in the Costa Rica Heart Study extended between 1994 and 2004 [42], we call for future research that accounts for the temperature variability to more accurately assess the independent mechanistic link between diet-attributable GHGEs, MI, and any other health outcomes when data collection occurs across the years.

In our study, the mean diet-attributable GHGEs were similar to the Chinese population in 2015 [57], but higher than previous studies conducted in European countries [16,17,21,22,23,53,54,55,74,75], China [56], and Japan [52]. We acknowledge the limitation of a crude comparison among countries because diet-attributable GHGEs are affected by dietary patterns, eating culture, and population characteristics. For example, a Spanish population with higher adherence to the Mediterranean diet had a lower mean of daily diet-attributable GHGEs of 3.0 (SD 0.94) kg CO_2_ eq. [22] compared to our study sample’s daily estimate of 11.0 (SD 4.77) and stratified by an MI status of 11.61 (SD 4.96) and 10.40 (SD 4.49) for cases and control, respectively. Furthermore, sample characteristics could be a source of variability in diet-attributable GHGEs estimations because higher values were reported among men than women [16,54,56,74] and our sample comprised 75% men due to the matching. In addition, the data source used to estimate the diet-attributable GHGEs has been suggested as a significant source of heterogeneity in evaluating diet-attributable emissions [52]. None of the previously mentioned studies used the same dataset as in the current study [49], which adds to the complexity of contrasting our estimate with previous studies. However, to put our finding into context of human activities, the annual diet-attributable GHGEs produced by the cases were approximately similar to the GHGEs of four direct return flights from Los Angeles, United States, to New York, United States (4.28 tons of CO_2_ eq.) [76]. 

Our study has several potential limitations. Despite using a validated FFQ to quantify intake among the Costa Rican population [45,77], a couple of inherited limitations should be acknowledged in assessing diet-attributable GHGEs. Firstly, self-reported dietary assessment is subject to measurement errors [78], and the possibility of differential measurement error in our dietary assessment due to the diagnosis of MI is highly plausible; however; we think that is less likely to be the case because the majority of participants reported that stress and smoking were the attributable case of MI and only a small percentage reported dietary intake being the cause [39]. Secondly, because diet-attributable GHGEs and energy intake are correlated [79], our estimates inherited self-reported intake-related errors. Nevertheless, previous studies have used diet history [21,22], FFQ [23,80], and 24 hr. recalls [17] to assess diet-attributable GHGEs as no validated diet-attributable GHGEs tool has yet been developed. We acknowledge that our dietary assessment was conducted between 1994 and 2004 and the dietary intake might not represent the current consumption among Costa Ricans, so further studies are needed to solidify the evidence. However, the dietary assessment was conducted during Costa Rican’s nutrition transition, which provides a time point reference for dietary intake during that time to compare diet-attributable GHGEs in future studies. Finally, our dietary assessment was limited to one year before the diagnosis of the first non-acute MI; however, cardiovascular disease has a long latency period, so repeated assessment and a longitudinal study design are needed to address the potential of changing lifestyle and dietary habits over many years. 

In our study, the assessment of the environmental impact of dietary intake is limited to the quantification of diet-attributable GHGEs. Although diet-attributable GHGEs are associated with other environmental domains [26] and are highly associated with health outcomes compared to cropland, nitrogen, phosphorus, and freshwater use [14], modifying diet-attributable GHGEs per se does not necessarily reduce other environmental impacts. For example, shifting to predominantly plant-based dietary patterns reduces diet-attributable GHGEs but increases water use [14,17]. Therefore, further research is needed to explore other environmental aspects to have a holistic picture of the dietary effect on our environment. Lastly, given the observational nature of our study, we acknowledge the possibility of residual confounding due to unmeasured or crudely assessed confounders.

## 5. Conclusions

Our study supports a positive association between diet-attributable GHGEs, driven by red meat consumption, and non-acute MI in a middle-income Latin American population undergoing the nutrition transition. These results supplement the evidence linking dietary intake with cardiovascular and planetary health based on the assessment of diet-attributable GHGEs. Also, our findings are in line with Chaudhary et al.’s proposed dietary modifications for Costa Rica toward a sustainable diet by reducing the diet-attributable GHGEs (−37%) via a reduction in beef consumption (−16%) [18]. Therefore, our public health message focuses on promoting a predominantly plant-based diet as a healthy and sustainable diet, while appraising food groups drivers of the diet-attributable GHGEs and considering other parameters of environmental impact. It is clear that a predominantly plant-based diet that includes a high proportion of black beans to rice will meet the essential protein and nutrient needs of the Costa Rican population. However, we acknowledge that red meat constitutes complete protein quality, with bioavailable minerals and essential nutrients [81]. Thus, more studies are needed to investigate the impact of a sustainable diet and health outcomes by incorporating not only community assets and resources such as food environments, food insecurity, food production and distribution, and other food system-related matters, but also by examining the gut microbiome and diet connection across different populations. Research is needed to explore Costa Rican attitudes, beliefs, and concerns about sustainable food choices to help facilitate and maintain traditional healthy and sustainable food practices. We also call on the importance of monitoring the dietary intake of populations affected by the nutrition transition given the documented meat consumption between 1995 and 2015 [29]. We support the use of new monitoring methods of dietary intake, such as food diary applications and smart devices, and the development of new models for analyzing diet-attributable GHGEs. 

## Figures and Tables

**Table 1:** Sociodemographic and lifestyle characteristics in cases of acute myocardial infarction and matched population-based controls ^a^ from the Costa Rica Heart Study (N = 3634).

	Controls N = 1817	Cases N = 1817	*p*-Value
	Mean ± SD	Mean ± SD	
Age, years	58 ± 11	59 ± 11	-
Female, %	25	25	-
Marital status, % married ^b^	73	66	<0.0001
Education, % post-secondary ^b^	15	13	<0.0001
Income, USD/month ^c^	569 ± 423	496 ± 394	<0.0001
Current smokers, % ^b^	21	40	<0.0001
Physical activity, METs/day ^c^	35 ± 16	34 ± 16	0.0039
Waist circumference, cm ^d^	91 ± 10	91 ± 9	0.4603
Diabetes, % ^b^	14	24	<0.0001
Hypertension, % ^b^	30	37	<0.0001
Total caloric intake, kcal ^d^	2393 ± 641	2532 ± 686	<0.0001
Total diet-attributable GHGEs, kg CO_2_ eq./year ^c^	3795 ± 1638	4238 ± 1811	<0.0001

^a^ Matched for age ± 5 years, gender, and area of residence; ^b^ McNemar test was used for binary variables; ^c^ Wilcoxon signed rank test was used when continuous variables were not normally distributed; ^d^ Paired *t*-test was used was used when continuous variables were normally distributed; Percentages are presented for categorical variables and mean ± standard deviations (SD) are presented for continuous variables. Abbreviations: METs: metabolic equivalents; GHGEs: greenhouse gas emissions; CO_2_: carbon dioxide; eq.: equivalent.

**Table 2:** Mean food group intake in cases of acute myocardial infarction and matched population-based controls ^a^ from the Costa Rica Heart Study (N = 3634).

	Intake (Serving/Year)			% of Total Diet-Attributable GHGEs (kg CO_2_ eq./year)		
	Controls	Cases	*p*-Value	Controls	Cases	*p*-Value
Red meat	426 ± 319	496 ± 373	<0.0001	44.85 ± 17.74	46.74 ± 17.87	0.0029
Fish and chicken	246 ± 145	260 ± 181	0.0546	8.81 ± 6.23	8.25 ± 5.95	0.0007
Dairy products	689 ± 534	753 ± 594	0.0027	12.09 ± 9.87	11.91 ± 9.96	0.2852
Eggs	199 ± 212	228 ± 259	0.0011	1.53 ± 1.85	1.57 ± 2.01	0.2916
Legumes	650 ± 406	679 ± 415	0.0145	1.08 ± 0.92	1.01 ± 0.83	0.0204
Grains, cereals, and starchy vegetables	3362 ± 1240	3531 ± 1337	0.0005	7.45 ± 3.66	6.97 ± 3.5	<0.0001
Vegetables	4726 ± 1757	4898 ± 1778	0.0010	1.84 ± 1.19	1.65 ± 1.03	<0.0001
Fruits	1053 ± 868	985 ± 769	0.0578	2.24 ± 2.21	1.91 ± 1.75	<0.0001
Sugar	1132 ± 898	1256 ± 1014	0.0008	7.84 ± 6.06	7.54 ± 6.52	0.0358
Unsaturated fat and oils	444 ± 353	442 ± 370	0.6331	0.62 ± 0.97	0.49 ± 0.73	<0.0001
Alcohol	154 ± 348	167 ± 417	0.9643	1.01 ± 2.39	0.98 ± 2.56	0.0868
Coffee	822 ± 516	947 ± 551	<0.0001	10.02 ± 7.95	10.34 ± 7.4	0.0340
Chocolate	37 ± 105	42 ± 130	0.5913	0.63 ± 1.76	0.63 ± 1.74	0.9111
Water, tea, and other beverages	1139 ± 753	1085 ± 753	0.0368	0 ± 0	0 ± 0	-

^a^ Matched for age ± 5 years, gender, and area of residence; Means ± standard deviations values are reported; Wilcoxon signed-rank test was conducted for all variables; Abbreviations: GHGEs: greenhouse gas emissions; kg: kilogram; CO_2_: carbon dioxide; eq.: equivalent.

**Table 3:** Distribution of the sociodemographic and lifestyle characteristics of the Costa Rican population-based controls by quintiles of diet-attributable GHGEs (N = 1817).

	Quintiles of Median Diet-Attributable GHGEs (kg CO_2_ eq./year) (min.–max.)				
	Q1 2002 (536–2418)	Q2 2780 (2419–3150)	Q3 3567 (3152–3992)	Q4 4453 (4002–5024)	Q5 5857 (5025–18,169)
**Marital status, % married**	18	20	21	21	19
**Education,% post-secondary**	16	20	24	19	21
**Income, USD/month**	335	411	484	503	503
**Current smokers, %**	17	18	18	22	24
Physical activity, METs/day	32	32	33	33	34
**Waist circumference, cm**	89	91	91	92	93
**Diabetes, %**	21	26	19	19	16
**Hypertension, %**	22	23	21	19	16
**Total energy intake, kcal/day**	1782	2100	2317	2596	3061

Median values are presented for continuous variables and percentages for categorical variables. Bolded variables are selected to be adjusted for in the final models. Abbreviations: METs: metabolic equivalents; GHGEs: greenhouse gas emissions; CO_2_: carbon dioxide; eq.: equivalent.

**Table 4:** Mean food group diet-attributable GHGEs among Costa Rican population-based controls (N = 1817).

Food Groups	Quintiles of Total Diet-Attributable GHGEs (kg CO_2_ eq./year) (Median)				
	Q1 1890	Q2 2779	Q3 3560	Q4 4477	Q5 6270
Red meat	512 ± 351	1096 ± 420	1622 ± 508	2413 ± 551	3815 ± 1320
Fish and chicken	224 ± 188	263 ± 178	313 ± 167	342 ± 169	385 ± 202
Dairy products	268 ± 233	377 ± 299	456 ± 359	472 ± 384	595 ± 434
Eggs	41 ± 58	46 ± 41	49 ± 47	58 ± 54	65 ± 69
Legumes	30 ± 23	35 ± 23	35 ± 24	37 ± 21	41 ± 22
Grains, cereals, and starchy vegetables	209 ± 65	231 ± 72	240 ± 72	261 ± 82	283 ± 73
Vegetables	48 ± 28	58 ± 31	59 ± 28	68 ± 35	79 ± 41
Fruits	60 ± 59	70 ± 60	77 ± 60	80 ± 63	88 ± 80
Sugar	165 ± 137	225 ± 177	296 ± 237	321 ± 228	411 ± 275
Unsaturated fat and oils	17 ± 26	19 ± 32	22 ± 29	23 ± 29	23 ± 28
Alcohol	17 ± 50	26 ± 69	44 ± 95	50 ± 101	54 ± 96
Coffee	286 ± 196	318 ± 195	324 ± 195	328 ± 202	389 ± 230
Chocolate	13 ± 37	17 ± 53	24 ± 67	26 ± 76	44 ± 99
Water, tea, and other beverages	0	0	0	0	0

Means (standard deviation) are presented. Abbreviations: METS: metabolic equivalents; GHGEs: greenhouse gas emissions; CO_2_: carbon dioxide; eq.: equivalent.

**Table 5:** Odds ratios (OR) and 95% confidence intervals (CI) by quintiles of total diet-attributable GHGEs in the Costa Rica Heart Study ^a^ (N = 3634).

	Quintiles of Total Diet-Attributable GHGEs (kg CO_2_ eq./year) (Median)					*p*-Trend	For Every 1000 kg CO_2_ eq./year Increases in Diet-Attributable GHGEs	*p*-Value
	Q1 2065 (n = 726)	Q2 2931 (n = 727)	Q3 3785 (n = 727)	Q4 4737 (n = 727)	Q5 6247 (n = 727)			
Basic model ^b^	1.00	1.08 (0.87, 1.34)	1.41 (1.14, 1.74)	1.60 (1.29, 1.99)	2.20 (1.78, 2.75)	<0.0001	1.17 (1.12, 1.22)	<0.0001
Multivariable model 1 ^c^	1.00	1.03 (0.82, 1.30)	1.35 (1.06, 1.71)	1.41 (1.09, 1.82)	1.81 (1.35, 2.43)	0.0001	1.12 (1.06, 1.18)	0.0001
Multivariable model 2 ^d^	1.00	0.96 (0.76, 1.22)	1.26 (0.99, 1.61)	1.30 (0.99, 1.70)	1.63 (1.20, 2.21)	0.0012	1.10 (1.04, 1.17)	0.0012

^a^ Cases and controls are matched for age ± 5 years, gender, and area of residence; ^b^ Adjusted for matching variables age, gender, and area of residence; ^c^ Basic model plus marital status, education, income, smoking, and total energy; ^d^ Multivariate model 1 plus diabetes, hypertension, and waist circumference; Abbreviations: GHGEs: greenhouse gas emissions; kg: kilogram; CO_2_: carbon dioxide; eq.: equivalent.

## Data Availability

Data are contained within the article.
